# Clinical relevance of oncogenic driver mutations identified in endometrial carcinoma

**DOI:** 10.1016/j.tranon.2021.101010

**Published:** 2021-01-12

**Authors:** Takafumi Watanabe, Hideaki Nanamiya, Manabu Kojima, Shinji Nomura, Shigenori Furukawa, Shu Soeda, Daisuke Tanaka, Takao Isogai, Jun-ichi Imai, Shinya Watanabe, Keiya Fujimori

**Affiliations:** a Department of Obstetrics and Gynecology, Fukushima Medical University, Fukushima 960-1295, Japan; b Translational Research Center, Fukushima Medical University, Fukushima 960-1295, Japan

**Keywords:** Endometrial carcinoma, Somatic mutations, Clinical molecular genetics, Prognostic biomarker

## Abstract

**Purpose:**

Endometrial carcinoma (EC) is a clinically heterogeneous disease characterized by a number of different histological subtypes, and its heterogeneity may be involved in the accumulation of multiple genetic alterations. The aim of this work was to investigate the comprehensive mutational profile of EC tumors, and examine the associations between somatic mutations and clinicopathological features or survival in EC patients.

**Methods:**

A total of 100 surgical tumors were obtained from EC patients who had previously undergone surgery. Genomic DNA samples extracted from fresh-frozen tissues were analyzed using the Ion AmpliSeq Cancer Hotspot Panel v2 Kit, covering 50 tumor-related genes.

**Results:**

Validated mutations were detected in 91 of the 100 tumors (91%) and identified in eight of the most frequently mutated genes, namely *PTEN* (57%), *PIK3CA* (51%), *TP53* (30%), *KRAS* (23%), *CTNNB1* (21%), *FBFR2* (13%), *FBXW7*(10%) and *RB1* (9%). *PTEN* mutations were found to associated with young age (< 60), early-stage, endometrioid histology, non-recurrence and better overall survival (OS). *CTNNB1* mutations were associated with young age, endometrioid histology and better OS. On the other hands, *TP53* mutations were associated with late-stage, non-endometrioid histology, high-grade, recurrence and worse OS. *FBWX7* mutations were associated with late-stage, vascular invasion and lymph node metastasis. *FGFR2* mutations correlated with deep (≥ 1/2) myometrial invasion.

**Conclusion:**

Our comprehensive mutational profile will be useful for understanding and evaluating the molecular characteristics of EC tumors, and may lead to the establishment of novel treatment strategies that improve the survival of patients with EC in the future.

## Highlights

• Comprehensive somatic mutations profiling in endometrial carcinoma.
• Relationships between somatic mutations and clinicopathological characteristics.
• Relatiohship between *FBXW7* mutation and vascular invasion in endometrial carcinoma.
• *FGFR2* mutations are related with deep myometrial invasion in endometrial carcinoma.

## Introduction

Endometrial cancer (EC) is the most frequently-reported malignancy of the female genital tract, and the seventh most commonly occurring cancer in women worldwide, as of 2018 [1]. EC often produces symptoms, such as postmenopausal or abnormal genital bleeding, at a relatively early-stage, so the disease is generally diagnosed early. Although more than 75% of EC patients are stage I at the time of diagnosis, and the 5-year overall survival (OS) rate for women with early-stage EC exceeds 80%, 10–15% of all patients experience recurrence [1,2]. Stage, histological type, tumor grade, myometrial invasion (MI), vascular invasion (VI) and lymph node metastasis (LNM) at the time of treatment are defined as independent prognostic factors in patients with EC by the International Federation of Obstetrics and Gynecology (FIGO) [3]. However, to explain EC heterogeneity with these clinicopathological factors alone is impossible [4]. Therefore, identification of new predictive markers for a high-risk phenotype would be very useful for selection of the most efficient therapies as well as for the development of novel treatment modalities.

Recently, comprehensive molecular profiling of cancer using next-generation sequencing (NGS) approaches has been increasingly used in oncology for diagnostic and therapeutic management decisions. Cancer-specific mutations, including single-nucleotide alterations and small insertions or deletions, are known to affect key driver genes early during tumorigenesis [5]. There are two types of EC: Type I EC (80–90%) characterized by an excess of estrogen is typically low-grade endometrioid endometrial carcinoma (EEC) and has a favorable outcome. In contrast, type II EC (10–20%) is usually non-endometrioid endometrial carcinoma (NEEC) (serous carcinoma, clear cell carcinoma and malignant mixed mullerian tumor) with high-grade tumors and poor prognosis. In previous reports, frequent somatic mutations of *PTEN, CTNNB1, PIK3CA, ARID1A* and *KRAS* in type I EC, as well as those of *TP53* genes in type II EC, have been identified using whole exome and genome sequencing analyses [6,7]. Although several studies have reported associations between each somatic mutation and the clinicopathological characteristics of EC [8], [9], [10], few studies have focused on a comprehensive somatic mutation analysis [6]. More recently, plasma circulating tumor DNA (ctDNA) has been reported to be effective in early diagnosis and therapeutic monitoring in human cancers [11]. As blood samples are easy to obtain multiple times on demand in a non-invasive method compared with standard tumor biopsy, somatic mutations become increasingly important as clinically useful biomarkers. In the present study, we performed a comprehensive mutational profile in EC patients using a cancer panel, and examined whether somatic mutation status is associated with risk factors or survival.

## Materials and methods

### Clinical samples

A total of 100 surgical specimens were obtained from EC patients who had undergone surgery at Fukushima Medical University Hospital between August 2013 and December 2017. The clinicopathological data for age, staging (FIGO 2008), histology, tumor grade (EEC), MI, VI, LNM, and recurrence were collected by operative reports, clinical notes and pathological reports. Histology was divided into EEC and NEEC, and adjuvant therapy was determined according to the physician's treatment strategy. The study design was approved by the ethics committee of Fukushima Medical University (No. 1953), and informed consent was obtained from all participants. All analyses were performed in accordance with the relevant guidelines and regulations.

### DNA extraction

Isolation and purification of genomic DNAs after extraction of RNAs from 100 frozen tumor samples were performed using ISOGEN reagent (Nippongene, Tokyo, Japan), according to the manufacturer's instructions. The concentration and quality of each DNA sample were assessed using NanoDrop One (ThermoFisher Scientific, Waltham, MA, USA).

### Somatic mutation detection

The Ion Ampliseq Cancer Hotspot Panel v2 on the Ion Torrent platform was used to detect 2790 mutations in 50 oncogenes and tumor suppressor genes [12,13]. In brief, 10 ng of genomic DNAs extracted from 100 frozen tumor samples were used to construct barcoded DNA libraries utilizing an Ion Ampliseq Library Kit 2.0 (Thermo Fisher Scientific). The obtained libraries were optimized using an Ion Library Equalizer Kit (Thermo Fisher Scientific), and then sequenced using an Ion Personal Genome Machine or Ion S5XL platform (Thermo Fisher Scientific). The sequencing reads were aligned to the reference genome build hg19, GRCh37, and converted into BAM files using Ion Torrent Suite software (Thermo Fisher Scientific). Sequence variants were then called using Ion Reporter 5.0 (Thermo Fisher Scientific), according to the manufacturer's instructions. The average sequencing depth reached at least 1500-fold per sample.

### Statistical analysis

The associations between somatic mutations and categorical variables, as well as between mutation rate or frequency and categorical variables, were evaluated using the chi-squared test or Mann–Whitney *U* Test. OS was evaluated as clinical outcomes. Survival distributions were calculated according to the Kaplan–Meier method, and statistical significance was determined using the log-rank test. Values of *P* < 0.05 were considered statistically significant. Statistical analysis of data was performed using SPSS version 25 software (SPSS, Inc., Chicago, IL, USA).

## Results

### Clinicopathological characteristics

The clinicopathological characteristics of the patients are shown in Table 1. A total of 100 EC patients who pervious undergone surgery were enrolled in this study. The median age at diagnosis was 62.5 years (range, 32–89 years), and 62 (62%) of the patients with high recurrence risk or advanced stage underwent platinum-based chemotherapy postoperatively. Among the 100 tumors, 82 were EEC and 18 were NEEC (nine serous, six clear cell, two undifferentiated and one mixed carcinoma). After a median follow-up of 37 months (range, 1–76 months), 78 (78%) patients were alive without clinical evidence of tumor. Recurrence was identified during the follow-up period in 22 (22%) patients: six (6%) patients were alive with disease; sixteen (16%) patients died due to their tumor between postoperative months 1 and 42 (median, 7.5 months).

**Table 1:** Clinicopathological characteristics of patients with endometrial cancer.

Characteristics	No. of cases	(%)
Age at diagnosis (years)		
< 60	45	(45)
≥ 60	55	(55)
Stage		
I	61	(61)
II	9	(9)
III	14	(14)
IV	16	(16)
Histology		
EEC	82	(82)
NEEC	18	(18)
Grade		
G1	54	(65.9)
G2	16	(19.5)
G3	12	(14.6)
Myometrial invasion		
> 1/2	54	(54)
≤ 1/2	46	(46)
Vascular invasion		
No	69	(69)
Yes	31	(31)
Lymph node metastasis		
No	84	(84)
Yes	16	(16)
Recurrence		
No	78	(78)
Yes	22	(22)

### Spectrum and frequency of mutations in EC

**Fig. 1:**
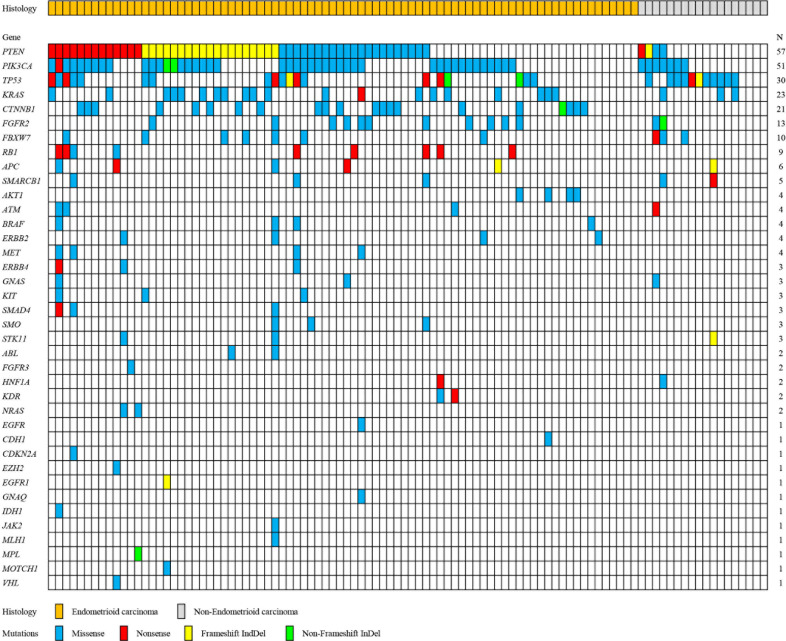
Summary of the relationships between somatic mutations and histological characteristics of endometrial cancer. All panels are aligned with vertical tracks representing 100 individuals.

In order to explore the spectrum and frequency of mutations in EC, 100 frozen tumors were analyzed. Fig. 1 shows a summary of somatically altered genes that are recurrently mutated. Validated mutations were detected in 91 of the 100 tumors (91%), and 77 tumors (77%) harbored concurrent mutations in two or more genes. Of 50 tumor-related genes, 38 were observed in EC tumors in the current study. Mutations were most frequently detected in *PTEN* (57%), *PIK3CA* (51%) and *TP53* (30%) (Fig. 1). *KRAS, CTNNB1, FBFR2, FBXW7* and retinoblastoma (RB) gene mutations were relatively frequent, with respective occurrence rates of 23%, 21%, 13%. 10% and 9%, followed by *APC* (6%); *SMARCB1* (5%); *AKT1, ATM, BRAF, ERBB2* and *MET*, (4%); *ERBB4, GNAS, KIT, SMAD4, SMO* and *STK11* (3%); *ABL, FGFR3, HNF1A, KDR* and *NRAS* (2%); and *EGFR, CDH1, CDKN2A, EZH2, EGFR1, GNAQ, IDH1, JAK2, MLH1, MPL, NOTCH1* and *VHL* (1%) (Fig. 1). A total of 284 (mean, 2.84) mutations were detected: 212 (74.6%) missense mutations; 39 (13.7%) nonsense mutations; 26 (9.2%) frameshift indels; and seven (2.5%) non-frameshift indels (Fig. 1).

**Fig. 2:**
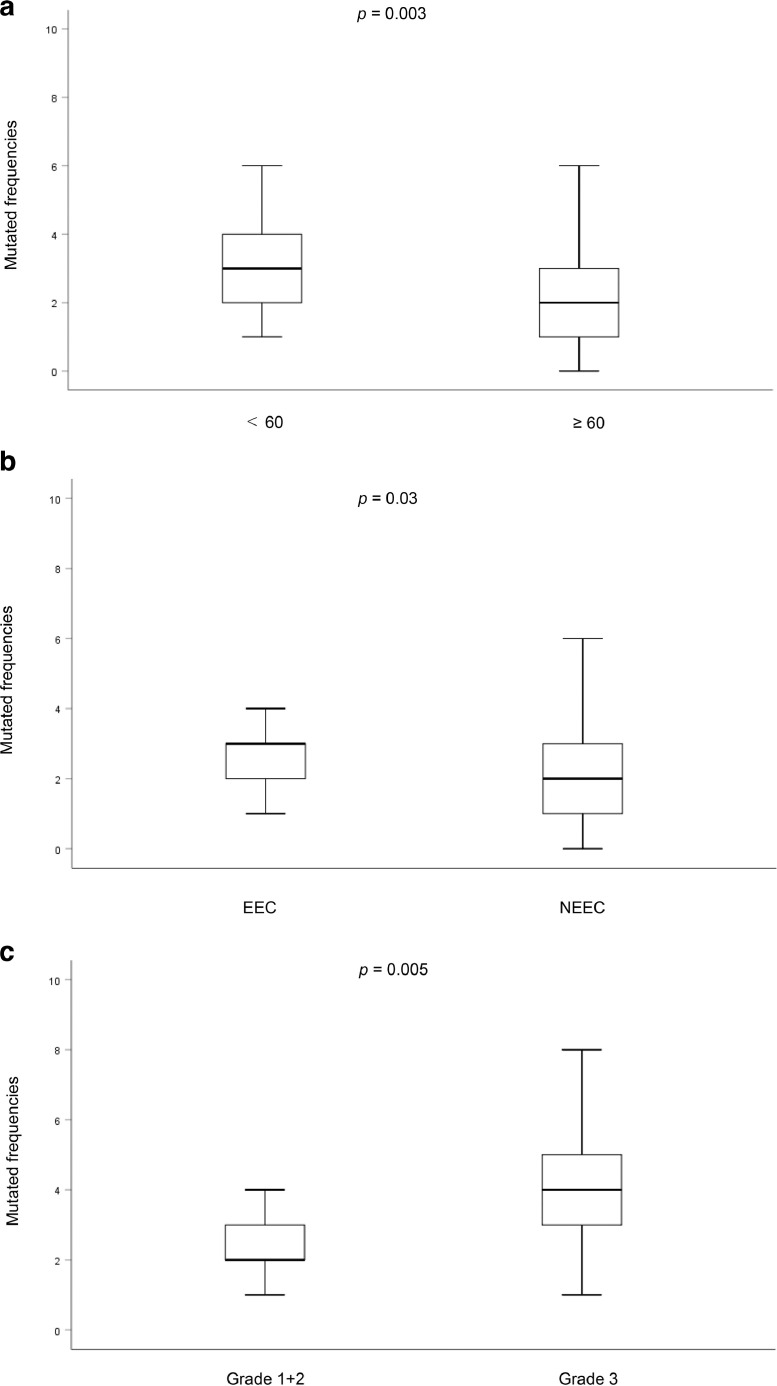
Box plot showing differences in mutation frequency between (a) < 60 and ≥ 60 (*P* = 0.003). (b) Grade 1 + 2 and Grade 3 (*P* = 0.005). (c) EEC and NEEC (*P* = 0.03). EEC: endometrial endometrioid carcinoma, NEEC: non-endometrial endometrioid carcinoma

We examined the differences in mutation rates between EEC and NEEC using a chi-squared test. The mutation rate was significantly higher in the EEC (93.9%, 77/82) than in the NEEC (77.7%, 14/18) (*P* = 0.03) (data not shown). The association between mutation frequencies and clinicopathological features was investigated using the Mann–Whitney *U* Test. Table S1 shows the mean mutation frequency with standard deviation and P-value. Age, tumor grade and histology were significantly associated with mutation frequencies (Fig. 2a–c). On the other hand, there were no significant associations between stage, MI, VI, LNM, or recurrence and mutation frequencies (Supplementary Table 1).

### Relationships between mutation status and clinicopathological characteristics

Table 2 shows a summary of the associations between eight most frequently mutated genes (*PTEN, PIK3CA, TP53, KRAS, CTNNB1, FBFR2, FBXW7* and *RB*) and clinicopathological characteristics in EC patients. Patients with *PTEN* and *CTNNB1* mutations had a significantly young age (< 60) than those without these mutations (*P* = 0.003 and *P* = 0.006, respectively). The *TP53* and *FBXW7* mutations were significantly observed in late-stage EC (*P* = 0.017 and *P* = 0.023, respectively), whereas the *PTEN* mutation was significantly more common in early-stage EC (*P* = 0.007). *TP53* mutation was significantly more common in the NEEC tumors (*P* = 0.001), whereas the *PTEN* and *CTNNB1* mutations were significantly more common in the EEC tumors (*P* = 0.001 and *P* = 0.02, respectively). *TP53* mutation was significantly associated with high-grade tumor (*P* = 0.0001). *FGFR2* mutation was significantly associated with in deep (≥ 1/2) MI (*P* = 0.016). On the other hand, the association of the *CTNNB1* mutation with superficial (< 1/2) MI was marginally significant (*P* = 0.071). *FBXW7* mutation was significantly associated with VI (*P* = 0.001), And the association between *TP53* mutation and VI was also marginally significant (*P* = 0.088). In contrast, the association between *CTNNB1* mutation and non-VI was marginally significant (*P* = 0.064), and *FBXW7* mutation was significantly associated with LNM (*P* = 0.029). The association of *TP53* mutation with LNM was also marginally significant (*P* = 0.056). On the other hand, the association of *PTEN* mutation with non-LNM was also marginally significant (*P* = 0.08). *TP53* mutation was significantly associated with recurrence (*P* = 0.004), whereas *PTEN* mutation was significantly associated with non-recurrence (*P* = 0.001).

**Table 2:** Frequency of *PTEN, PIK3CA, TP53, KRAS, CTNNB1, FGFR2, FBXW7*, and *RB* mutations according to demographic and clinicopathological characteristics.

Gene	PTEN			PIK3CA			TP53			KRAS		
	Wt	Mut	*P* value	Wt	Mut	*P* value	Wt	Mut	*P* value	Wt	Mut	*P* value
Characteristic	*n* = 43	*n* = 57		*n* = 49	*n* = 51		*n* = 70	*n* = 30		*n* = 77	*n* = 23	
Age												
< 60	12	33	**0.003**	19	26	0.22	31	14	0.83	35	10	0.87
≥ 60	31	24		30	25		39	16		42	13	
Stage												
I/II	24	46	**0.007**	33	37	0.57	54	16	**0.017**	52	18	0.32
III/IV	19	11		16	14		16	14		25	5	
Histology												
EM	29	53	**0.001**	38	44	0.26	63	19	**0.001**	62	20	0.48
Non-EM	14	4		11	7		7	11		15	3	
Grade												
G1/G2	26	44	0.42	35	35	0.11	59	11	**0.0001**	51	19	0.16
G3	3	9		3	9		4	8		11	1	
Myometrial invasion												
< 1/2	22	32	0.62	26	28	0.85	33	13	0.73	41	13	0.78
≥ 1/2	21	25		23	23		37	17		36	10	
Vascular invasion												
No	27	42	0.24	35	34	0.61	52	17	0.088	55	14	0.34
Yes	16	15		14	17		18	13		22	9	
Lymph node metastasis												
No	33	51	0.08	42	42	0.64	62	22	0.056	65	19	0.83
Yes	10	6		7	9		8	8		12	4	
Recurrence												
No	27	51	**0.001**	38	40	0.91	60	18	**0.004**	59	19	0.54
Yes	16	6		11	11		10	12		18	4	


	CTNNB1			FGFR2			FBXW7			RB		
	Wt	Mut	*P* value	Wt	Mut	*P* value	Wt	Mut	*P* value	Wt	Mut	*P* value
	*n* = 79	*n* = 21		*n* = 87	*n* = 13		*n* = 90	*n* = 10		*n* = 91	*n* = 9	
Age												
< 60	30	15	**0.006**	39	6	0.93	40	5	0.73	39	6	0.17
≥ 60	49	6		48	7		50	5		52	3	
Stage												
I/II	52	18	0.077	63	7	0.17	66	4	**0.023**	64	6	0.82
III/IV	27	3		24	6		24	6		27	3	
Histology												
EM	62	21	**0.02**	71	11	0.79	75	7	0.29	73	9	0.14
Non-EM	17	0		16	2		15	3		18	0	
Grade												
G1/G2	50	20	0.14	61	9	0.72	65	5	0.28	64	6	0.093
G3	11	1		10	2		10	2		9	3	
Myometrial invasion												
< 1/2	39	15	0.071	51	3	**0.016**	51	3	0.11	48	6	0.42
≥ 1/2	40	6		36	10		39	7		43	3	
Vascular invasion												
No	51	18	0.064	62	7	0.21	67	2	**0.001**	62	7	0.55
Yes	28	3		25	6		23	8		29	2	
Lymph node metastasis												
No	65	19	0.36	73	11	0.94	78	6	**0.029**	77	7	0.59
Yes	14	2		14	2		12	4		14	2	
Recurrence												
No	59	19	0.12	68	11	0.55	72	6	0.14	71	7	0.91
Yes	20	2		20	2		18	4		20	2	

Significant *P* values (*P* < 0.05) were shown in bold style.

### Association of mutation status with clinical survival

**Fig. 3:**
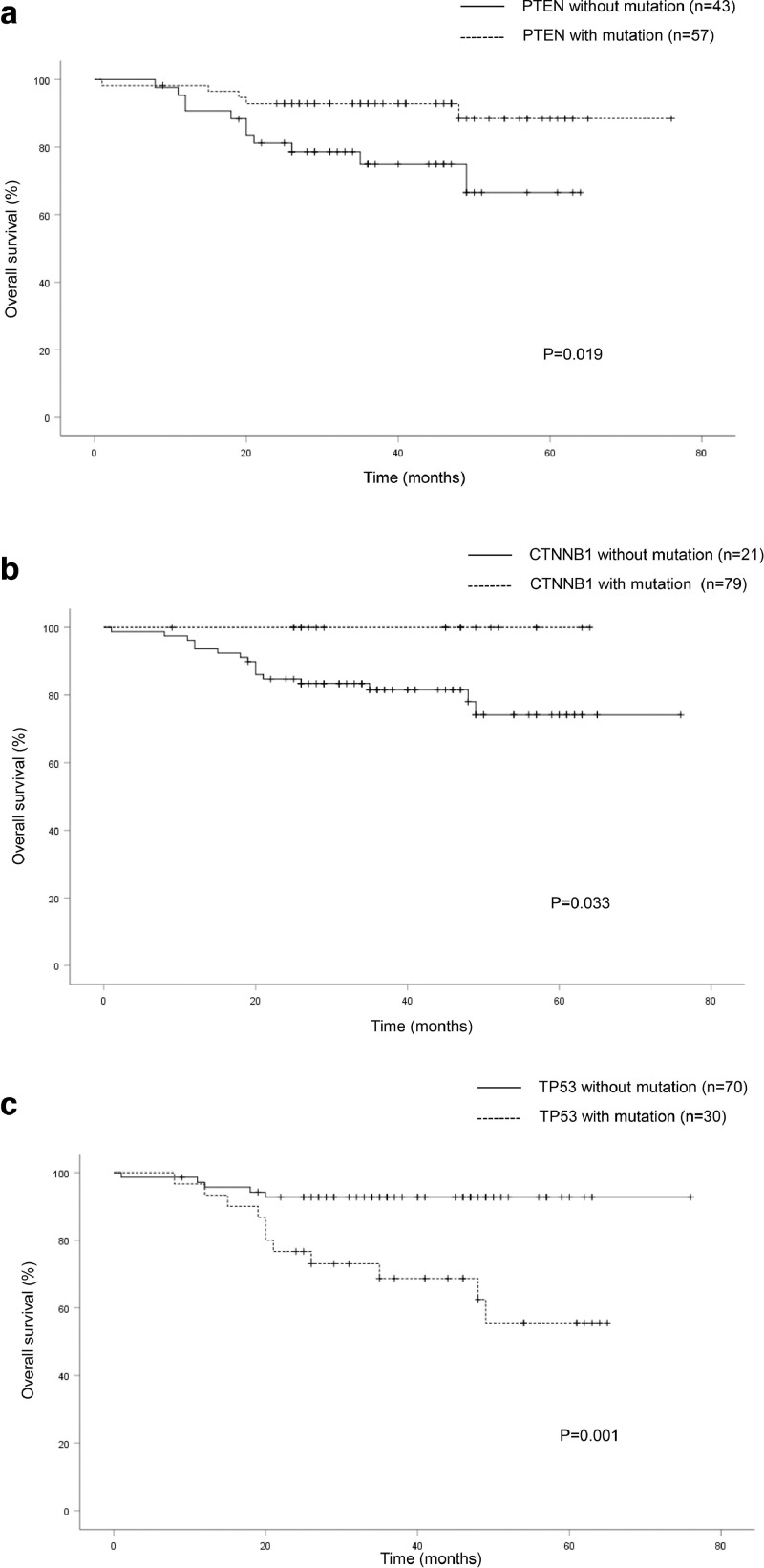
The Kaplan–Meier curves of overall survival in patients with endometrial cancer. (a) Patients with and without *PTEN* mutations (*P* = 0.019). (b) Patients with and without *CTNNB1* mutations (*P* = 0.033). (c) Patients with and without *TP53* mutations (*P* = 0.001).

The relationships between the eight most frequently mutated genes (*PTEN, PIK3CA, TP53, KRAS, CTNNB1, FBFR2, FBXW7* and *RB1*) and clinical survival were analyzed by log-rank test. The Kaplan–Meier curves for *PTEN, TP53* and *CTNNB1* mutations in the EC patients are shown in Fg. 3. The patients with *PTEN* and/or *CTNNB1* mutations had a significantly better OS than those without these mutations (*P* = 0.019 and *P* = 0.033, respectively) (Fig. 3a and b), whereas those with *TP53* mutation had a significantly worse OS (*P* = 0.001) (Fig. 3c). However, *PIK3CA, KRAS, FBFR2, FBXW7* and *RB1* mutations had no significant associations with OS (data not shown).

## Discussion

Our findings showed a very high rate (91%) of a potential oncogenic driver or drug targetable mutations in EC patients. Validated mutations have been also reported in 78–94% of EC patients using a cancer panel [14,15], and EC is known as a tumor with a high frequency of mutated genes [16]. A comprehensive genomic analysis by The Cancer Genome Atlas (TCGA) resulted in the molecular classification of EC into four distinct subgroups [6]: the DNA polymerase epsilon (POLE) group, which has an exceedingly high mutation rate in conjunction with somatic mutations in the exonuclease domain of *POLE*; the microsatellite instability (MSI) group, which has a high mutation rate and is a hallmark of a defective mismatch repair system because of hypermethylation of the MLH1 promoter or a germline mutation in one of the mismatch repair genes; the copy number (CN)-low group, which has a low mutation rate and most of the microsatellite stable EEC; and the CN-high group, which has a low mutation rate and consists serous carcinoma with extensive high-level CN alterations and frequent somatic mutations in *TP53*. POLE, MSI and CN-low are almost exclusively of endometrioid morphology, whereas serous carcinoma contained in NEEC is almost exclusively in the CN-high group. POLE and MSI tumors have high mutation frequencies, which are approximately 100-fold and six-fold greater, respectively, than those seen in CN-low or CN-high tumors. POLE-mutated tumors are frequently of high-grade EEC. In the present study, the mutated frequencies were significantly higher in high-grade EEC tumors than in the low-grade EEC tumors. Moreover, the EEC tumors had a significantly higher mutation frequency than the NEEC tumors. Although we did not examine integrated *POLE* mutation, or perform MSI assay or CN analysis, our findings were consistent with those of TCGA.

We here investigated the associations between the eight most frequently mutated genes and the prognostic risk factors for survival in EC patients. *PTEN* is identified as a tumor suppressor that is mutated in a large number of cancers at high frequency. *PTEN* was found to be mutated at the highest frequency in the present study. In particular, the rate of *PTEN* mutations in the EEC tumors was significantly higher than that in the NEEC tumors. The difference in the prevalence of *PTEN* mutation between EEC and NEEC was similar to those reported in previous studies [6,17]. Since *PTEN* mutation is present in a relatively large proportion of atypical endometrial hyperplasia, which is known as a precancerous lesion, it may be considered an early event in the pathogenesis of EEC tumor [18,19]. *PTEN* mutation is frequently observed in type I EC and is associated with young age, early-stage, endometrioid subtype, low-grade and favorable prognosis as suggested by previous studies [20,21], similar to our results.

*PIK3CA* is one of the most commonly mutated genes in solid cancers, and the majority of *PIK3CA* mutations cluster in hotspot regions in exon 9 (the helical domain) and exon 20 (the kinase domain) [22]. The PIK3CA mutation has been frequently detected in not only EEC but also NEEC [23,24] as in the present study. As for survival, no significant association has been reported between *PIK3CA* mutations and disease-free survival in 94 EC patients [10]. On the other hand, associations have been reported between *PIK3CA* missense mutation and unfavorable outcome in grade 3 EEC tumors, and between *PIK3CA* exon 9 mutations and reduced survival in EC patients [25,26]. In the current study, there were no significant associations between the *PIK3CA* mutation and any clinicopathological features or survival. A consensus on the mechanism by which *PIK3CA* affects EC prognosis has yet to be achieved. Evaluation of the relationship between *PIK3CA* mutation and risk factors requires individualization with large samples.

Tumor suppressor p53 plays an important role in the preservation of genomic stability from various damages through the regulation of cell-cycle checkpoints, DNA repair, senescence, and apoptosis; furthermore, *TP53* is one of the most frequent alterations in human cancer. The frequency of somatic mutation in *TP53* was 30% in our study. In particular, *TP53* mutations in the EEC tumors were significantly fewer than those in the NEEC tumors, and the proportion was similar to that of a previous study [27]. In addition, the associations of *TP53* mutation with late-stage, high grade, and poor OS were statistically significant. Furthermore, *TP53* mutations had marginally significant associations with LNM. The CN-high category represents serous-like EC, and mostly incorporates *TP53* mutation, indicating a poor prognosis [6]. These results suggest that somatic mutation in *TP53* may be a good predictive biomarker for poor prognosis in EC patients, and has the opposite characteristic of *PTEN* mutation.

The *KRAS* proto-oncogene regulates cell division as a result of its ability to relay external signals to the cell nucleus. Activating mutations in the *KRAS* gene promotes down-regulation of MAPK or PI3K/AKT, which further results in excessive cell proliferation and subsequent carcinogenesis. *KRAS* mutation is present in up to 25% of all human tumors, and this is one of the most frequently activated oncogenes [28]. *KRAS* mutations in EC have been mostly associated with type I estrogen-dependent EC, and their frequency is estimated around 10–30% [29], [30], [31]. Although *KRAS* mutation was also found in 24% (20/82) of the EEC tumors, there was no significant difference between EEC and NEEC in the present study. *KRAS* mutation is a relatively common event in endometrial carcinogenesis, but its prognostic value is limited. Sideris et al reported that a consensus on the exact way that *KRAS* overall affects EC prognosis has yet to be achieved [32]. In the present study, no significant differences between *KRAS* mutation and OS was observed, so the prognostic significance of *KRAS* mutation remains controversial.

*CTNNB1* is a gene involved in the Wnt signaling pathway, which regulates cell growth, motility and differentiation. *CTNNB1* mutation is the activation of the Wnt signaling pathway, and has been specifically shown to be associated with carcinogenesis in many types of tumors [33]. Since *CTNNB1* mutations seemingly occur in atypical endometrial hyperplasia, *CTTNB1* mutation plays a critical role in the initiation and early progression of EEC tumors [34]. Other studies reported an increase in *CTNNB1* mutations in EEC compared to NEEC [35,36], and no *CTNNB1* mutation was observed in the NEEC tumors in the present study. *CTNNB1* mutation was detected in 25.6% (21/82) of the EEC tumors, which was similar to the result of previous reports [35], [36], [37]. We here demonstrated that *CTNNB1* mutation was significantly associated with endometrioid subtype and favorable OS, and was borderline significantly associated with young age, early-stage, superficial MI and VI. The clinicopathological features of *CTNNB1* mutations were similar to those of PTEN mutations; 90% (19/21) of tumors with *CTNNB1* mutation also had *PTEN* mutation. These data suggest that *CTNNB1* mutation may be a favorable prognostic biomarker in addition to *PTEN* mutation. However, recent studies have described significant associations between *CTNNB1* mutations and poor outcome among low-risk EEC patients, suggesting prognostic significance of *CTNNB1* mutation [37], [38], [39], [40]; thus, the clinical significance of *CTNNB1* mutation among low-risk EEC tumors needs to be carefully investigated.

*FGFR2* is one of the receptors for fibroblast growth factor, and has been shown to be activated in a number of cancers through a variety of mechanisms, including gene amplification, translocations, and point mutations [41]. Some studies subsequently reported a *FGFR2* mutation frequency of 10–16% in EC tumors [36,42,43], and said frequency in the current study was 13%. In the present study, there was a significant association between *FGFR2* mutation and deep MI. To the best of our knowledge, these findings have not yet been reported. Jeske et al. suggested that clinical trials testing the efficacy of FGFR inhibitors in the adjuvant setting to prevent recurrence and death are warranted because *FGFR2* mutations are associated with poor outcomes in EEC [43]. Although we did not investigate the relationships between *FGFR2* mutations and OS, *FGFR2* mutations may contribute to a poor prognosis for EC with MI.

*FBXW7* is a critical tumor suppressor that regulates proteasome-mediated degradation of various oncoproteins, such as cyclin E, c-Myc, Mcl-1, mTOR, Jun, Notch and AURKA in human cancer [44]. *FBXW7* mutations have been reported in several types of human cancers and found in 2.54% across all human tumors, according to COSMIC database meta-analyses [45]. Previous studies subsequently reported an *FBXW7* mutation frequency of 2–16% in EC tumors [30,46], and said frequency in the current study was 10%. In particular, *FBXW7* mutation has been observed in 20–30% of cases of serous EC [6,23,24] and was also found in 30% (3/10) in the present study (data not shown). The results of the present study showed that *FBXW7* mutation associated with late-stage, VI and LNM. In particular, the relationship between *FBXW7* mutation and VI in EC was a novel finding. Loss of FBXW7 function caused by *FBXW7* mutation resulted in high Brg1 expression, and was consequently associated with VI, LNM and distant metastasis in gastric cancer [47]. Although there was no significant association between *FBXW7* mutation and OS in the current study, *FBXW7* mutation may be a predictive biomarker for poor prognosis.

The *RB1* gene, located on chromosome 13, is a well-known tumor suppressor gene that was discovered in genetic studies of hereditary retinoblastoma [48]. Defects in this gene are a cause of childhood cancer, bladder cancer, and osteogenic sarcoma. Loss of heterozygosity (LOH) was reported in 18% of *RB* genes in EC and pRB downregulation was consistent with LOH [49]. Although *RB* mutation was detected in 9% of EC patients, no significant association was observed between clinicopathological features or survival and *RB* mutation. Further studies are needed with larger study populations to evaluate whether the inactivation of *RB1* is a useful biomarker.

However, the present study must be interpreted with caution, and a few limitations should be kept in mind. Said limitations include the small sample size, heterogeneity of histology or treatment within the cohort, and short follow-up period as they may have affected the associations between the somatic mutations and clinical outcomes. A subgroup analysis using a larger sample size would likely provide more definitive results. Another limitation is that our analysis relied on sequencing with a cancer hotspot panel, and only explained a portion of the total genetic alterations. The NGS platform used in this study detected only single nucleotide variants, and thus it was impossible to detect copy number variants and MSI status. Therefore, a large number of samples with long-term follow-up need to be prospectively analyzed using whole exome or genome sequencing that can detect single number variants, copy number variants and MSI status. Furthermore, other novel genetic or epigenetic alterations should be explored as well.

In summary, we here demonstrated that our comprehensive NGS analysis using a cancer panel was feasible for mutational profiling of EC tumors. Although whole exome and genome sequencing are useful methods for detecting somatic mutations in human cancers, they are difficult to perform in clinical practice because of problems regarding scalability and time- and cost-effectiveness. Therefore, it is necessary to provide a reduced set of the most informative diagnostic and prognostic clinical markers. Although the mutated gene significance for clinical outcomes in EC patients was similar to that found in previous work, new findings, the association between *FGFR2* mutation and deep MI and that between *FBXW7* mutation and VI, were revealed. The current study suggests that EC patients can benefit from molecular profiling with predictable prognostic factors and select systemic adjuvant chemotherapy in combination with specific targeted therapies. We believe that this work will be useful for understanding and evaluating the molecular characteristics of EC patients, and may lead to the establishment of novel treatment strategies that improve the survival of patients with EC in the future.

## Authorship contribution statement

Takafumi Watanabe: Conceptualization, Methodology, Investigation, Writing - Original Draft. Hideaki Nanamiya, Daisuke Tanaka, Takao Isogai and Jun-ichi Imai: Data curation, Formal analysis. Manabu Kojima, Shinji Nomura, Shigenori Furukawa and Shu Soeda: Resources, Investigation. Shinya Watanabe: Project administration, Funding acquisition. Keiya Fujimori: Supervision Writing - Review & Editing.

## Ethics approval and consent to participate

The study protocol was approved by the Ethics Committee of Fukushima Medical University School of Medicine (No. 1953), and informed consent was obtained from all patients. All participants signed informed consent forms.

## Declaration of Competing Interest

The authors declare that they have no competing interests.

## Appendix Supplementary materials

Image, application 1
