# Supplementary material for: Clinical relevance of oncogenic driver mutations identified in endometrial carcinoma
Source: Transl Oncol. 2021 Jan 12;14(3):101010. doi: 10.1016/j.tranon.2021.101010 (PMC7810788; doi:10.1016/j.tranon.2021.101010)
Supplement: Supplementary file 1 [file mmc1.docx]

**Supplementary Table 1** Summary of the associations between mutation frequencies and clinicopathological features in patients with endometrial cancer.

|  | Mean | s.d. | P value |
| --- | --- | --- | --- |
| Age at diagnosis (years) |  |  |  |
| < 60 | 3.44 | 2.27 | 0.003 |
| ≥ 60 | 2.34 | 2.11 |  |
| Stage |  |  |  |
| I/ II | 2.66 | 2.04 | 0.246 |
| III/IV | 3.27 | 2.63 |  |
| Histology |  |  |  |
| EM | 3.02 | 2.3 | 0.03 |
| Non-EM | 2 | 1.79 |  |
| Grade |  |  |  |
| G1/G2 | 2.73 | 2.01 | 0.005 |
| G3 | 4.75 | 3.03 |  |
| Myometrial invasion |  |  |  |
| > 1/2 | 2.74 | 2.22 | 0.536 |
| ≤ 1/2 | 2.96 | 2.27 |  |
| Vascular invasion |  |  |  |
| No | 2.65 | 2.14 | 0.142 |
| Yes | 3.25 | 2.42 |  |
| Lymph node metastasis |  |  |  |
| No | 2.78 | 2.33 | 0.269 |
| Yes | 3.12 | 1.79 |  |
| Recurrence |  |  |  |
| No | 2.88 | 2.28 | 0.763 |
| Yes | 2.68 | 2.14 |  |
